# Analysis of selected T cell subsets in peripheral blood after exhaustive effort among elite soccer players

**DOI:** 10.11613/BM.2018.030707

**Published:** 2018-10-15

**Authors:** Dorota Kostrzewa-Nowak, Robert Nowak

**Affiliations:** 1 Department of Biological Bases of Physical Education, Faculty of Physical Education and Health Promotion, University of Szczecin, Szczecin, Poland; 2 Centre for Human Structural and Functional Research, Faculty of Physical Education and Health Promotion, University of Szczecin, Szczecin, Poland

**Keywords:** athletes, fatigue, flow cytometry, soccer, T-lymphocytes

## Abstract

**Introduction:**

Physical exercise induces developing of naïve T lymphocyte subsets into polarised effector ones by immune system. The aim of the study was to examine the influence of exhaustive effort on the selected Th cell subsets and inflammation markers among soccer players.

**Materials and methods:**

Fourteen soccer players aged 18 (16-21) years old performed the progressive efficiency test on mechanical treadmill. Th and Tc memory lymphocytes’ subsets and selected cytokine concentrations pre-exercise, post-exercise and in recovery were analysed by flow cytometry.

**Results:**

Physical effort induced changes in Th cell percentages. Increase in recovery Treg and Th17 cell subsets’ percentages in comparison to pre-exercise values were observed (10.98 (9.83-14.07) *vs.* 3.95 (3.15-5.53), P < 0.001 in autumn; 10.58 (7.54-12.67) *vs.* 4.83 (3.73-6.81), P < 0.010 in spring for Treg and 29.21 (26.34-32.16) *vs.* 21.64 (18.48-25.76) in autumn; 27.15 (24.60-29.16) *vs.* 17.43 (15.83-19.77) in spring, both P < 0.010; for Th17, respectively). Increases in Th1 cell percentages post-exercise (31.86 (28.72-33.72) in autumn, 25.60 (21.50-29.19) in spring, both P < 0.010) and in recovery (34.64 (31.21-38.20) in autumn; 26.68 (25.17-28.07) in spring, both P < 0.001) compared to pre-exercise (22.70 (21.21-26.74) for autumn and 15.64 (14.38-19.63) for spring, respectively) were found. Interestingly, no changes in Th2 cell subsets were found. Post-exercise and recovery changes in IL-6, IL-8, TNF-α and IL-10 were also observed.

**Conclusions:**

It seems that the given effort in the progressive test induced an anabolic effect being related not only with cytokine profile but also with CD4^+^ T cells’ differentiation and peripheral distribution.

## Introduction

Various biochemically- and physiologically-based neuromotor changes play a vital role in biological adaptation of highly qualified athletes to the physical effort (*1*-*3*). Immune alterations may provide to the reduction in capacity and endurance levels in elite athletes by *e.g.* increased susceptibility to infections. During exercise, inflammatory environment arises, stimulating B and T lymphocytes to proliferate and differentiate respective to local demand. When the inflammatory agents, as well as these cells are eliminated, memory cells remain and proliferate to ensure rapid immune response in similar circumstances in the future (*4*). Moreover, in response to pathological (*e.g.* infection) or semi-pathological (*e.g.* physical exercise) state, the innate immune system leads to T cell activation on one hand, and the development of naïve T lymphocyte subsets into polarised effector ones on the other (*5*). It is well known that interferon gamma (INFγ), tumour necrosis factor α (TNF-α) and interleukin 2 (IL-2) produced by Th1 cells are responsible for stimulation of Tc cell-associated immune response. On the other hand, it is well described that Th2 cells can release interleukins (*e.g.* IL-6, IL-10) related with B cells’ growing and differentiation (*5*-*7*). Th17 cells are also classified as CD4^+^ subset synthetizing interleukins (*e.g.* IL-6) and releasing other signalling factors, including TNF-α (*8*, *9*). The presence of Th17 cells in peripheral blood is related to chronic inflammation and autoimmune diseases (*7*, *9*, *10*).

The plasticity of effector CD4^+^ T cell subsets is of high importance in triggering multidirectional immune response processes being responsible for *e.g.* pathogen elimination, induction of humoral response or limitation of immunopathological states, so it may also be of high importance in the response to the physical effort (*11*).

While there were numerous data describing the change between Th1 and Th2 balance and post-effort cytokine profile in young and old sedentary subjects, runners, walkers, cyclists, triathletes, marathon runners, and rowers over past two decades, there were no investigations describing changes in soccer players (*5*, *12*-*14*). Based on literature data, it can be stated that high intensity exercise favours emerging of Type 2 (T2, including Th2 and Tc2) cell subsets, being responsible for down-regulation of post effort anti-viral protection (*5*, *15*). On the other hand, it is well known that an adequate training programme performed during the training in each phase of the season causes disorder of homeostasis among athletes. Moreover, exercise-induced muscle damage can result in a release of inflammatory signalling molecules and myocellular enzymes into the blood. Although there are numerous immunological studies describing the molecular mechanisms of immune responses on different antigens and pathogens, the impact of intensive physical effort on the CD4^+^ T cell subsets is not fully understood. There is still a need to search for answers regarding mechanisms responsible for increasing athletes’ physical capacity as well as induction of exercise-induced immunosuppression processes. We hypothesise that CD4^+^ T cells are of key importance in immune response to the physical effort of high intensity. Therefore, the main aim of this study was to examine the influence of exhaustive effort on the selected CD4^+^ T cell subsets and inflammation markers among soccer players which were determined before, just after the progressive test and during recovery time before spring and autumn competition rounds.

## Materials and methods

### Study design

This is an experimental study performed in Physiology as well as Biochemistry Laboratories, Centre for Human Structural and Functional Research, Faculty of Physical Education and Health Promotion, University of Szczecin, Szczecin, Poland.

All participants of the study belong to the same sport club and participated in the same annual macrocycle training plan. It must be pointed out that the year-long training macrocycle starts in autumn. Moreover, all laboratory tests were conducted at the same time for each participant. Both experiments were performed after two weeks of the summer or winter club vacation time (in July 2017 and January 2018, respectively), when the participants were asked to refrain from physical effort, especially training units. To analyse the impact of maximal effort on differentiation and distribution of selected T cell subsets, CD4^+^ and CD8^+^ T lymphocytes’ subsets, namely naïve, central and effector memory ones before (pre-exercise), immediately after the progressive test until exhaustion (post-exercise), and during recovery time (recovery; about 17 hours after the test) were determined.

### Subjects

Fourteen elite male soccer players aged 18 (16-21) years old with at least 9 years of training experience belonging to Pogoń Szczecin S.A., a top league soccer club, were recruited in the study. All soccer players qualified to this study were playing in a midfielder, striker or defender position during the experiment time. We excluded goalkeepers form the experiment because of different training loads.

Participants had no history of any metabolic syndrome (according to International Diabetes Federation description: diabetes, prediabetes, abdominal obesity, high cholesterol and high blood pressure) or cardiovascular diseases (defined by World Health Organization as disorders of the heart and blood vessels) (*16*, *17*). They were non-smokers and refrained from taking any medications or supplements known to affect metabolism. Since the exercise protocol is routinely performed by the soccer club, the recruitment of the participants consisted of informing them (and their parents, where appropriate) about the study and inviting them to take part in it by donating extra blood samples. Participants (and their parents, where appropriate) were fully informed of any risks and discomfort associated with the experimental procedures before giving their written consent to participate. The study was approved by the Local Ethics Committee in accordance with the Helsinki Declaration.

All athletes not meeting the inclusion criteria (*e.g.* not giving us or later withdrawing the consent to participate, goalkeepers) or participants who did not take part in the second experiment were excluded from the study.

### Methods

Participants’ body mass and body composition parameters (body mass index (BMI), basal metabolic rate (BMR), percentage of fat (FAT), fat free mass (FFM), total body water (TBW)), were determined using Body Composition Analyzer Tanita BC-418MA (Tanita, Tokyo, Japan).

All the participants performed the same progressive efficiency test on mechanical treadmill until exhaustion. It started with 5 minutes of warm-up running with the speed of 5 km/h. During the proper test the speed increased by 2 km/h after each 3 minutes of the test until exhaustion, it means until the athlete was unable to continue the run (achieving each individual’s maximum fatigue). The cardiorespiratory fitness measures: maximum oxygen uptake (VO_2_max), maximum ventilation (VE), anaerobic threshold (AT), respiratory quotient (RQ), respiratory compensation (RC), maximal voluntary ventilation (MVV), metabolic equivalent (MET) and respiratory frequency (Rf) were determined using state-of-the-art breath by breath gas exchange data analyser Quark CPET (Cosmed, Albano Laziale, Italy) (*18*). Additionally, every 3 minutes of the exercise, before increasing the workload (treadmill speed), finger capillary blood was taken for determination of lactate (LA) concentration in order to establish athlete’s anaerobic threshold. It must be pointed out that, according to the soccer club procedure, the fingertip blood samples were taken during the run, since the athletes are used to it. The LA concentrations were determined using mobile blood lactate monitoring system (THE EDGE Lactate Analyser, Apex Biotechnology Copr., Hsinchu, Taiwan).

During both experiments, blood samples were obtained tree times from the elbow vein: before the test (pre-exercise), no longer than 5 minutes after the test (post-exercise) and about 17 hours after the test, at the end of recovery time (recovery). The value of about 17 hours of recovery represents the mean longest period between two physical efforts, *e.g.* between a soccer match and the next training or between two trainings.

Each time, blood samples were taken into 7.5 mL S-Monovette tube with ethylenediaminetetraacetic acid (K_3_EDTA, 1.6 mg EDTA/mL blood) (SARSTEDT AG & Co., Nümbrecht, Germany). All analyses were performed immediately after blood collection.

Total white blood cells (WBC) and lymphocytes (LYM) counts were analysed using haematology analyser ABX Micros 60 (Horiba ABX, Warsaw, Poland).

All flow cytometric analyses were performed using BD Accuri™ C6 flow cytometer (Becton Dickinson, Franklin Lakes, USA) and the results of flow cytometric data were calculated using BD Accuri™ C6 software (ver. 1.0.264.21).

Lymphocyte T, Th, and Tc subsets phenotyping in erythrocyte-lysed blood samples was performed using BD Multitest™ IMK kit (BD Biosciences, San Jose, CA, USA) according to the manufacturer’s protocol. The antibodies used in the assay included: FITC-labelled CD3, PE-labelled CD8, PerCP-labelled CD45, and APC-labelled CD4. For each sample, the fluorescence signal of at least 10^4^ ungated events was measured.

To determine Th1 and Th2 cell subsets in erythrocyte-lysed blood samples, Human Th1/Th2/Th17 Phenotyping Kit (BD Biosciences, San Jose, USA) was used according to the manufacturer’s protocol. The antibodies cocktail contained: FITC-labelled interferon-gamma, PerCP-Cy5.5-labelled CD4, and APC-labelled IL-4. For each sample, the fluorescence signal of at least 10^4^ events gated for the forward and side light-scatter characteristics of lymphocytes was measured.

Lymphocyte Th17 and Treg subsets determination in erythrocyte-lysed blood samples was performed using HumanTh17/Treg Phenotyping Kit (BD Pharmingen™, San Jose, USA) according to the manufacturer’s protocol. The antibodies cocktail contained: PE-labelled IL-17A, PerCP-Cy5.5-labelled CD4, and Alexa Fluor® 647-labelled FoxP3. For each sample, the fluorescence signal of at least 10^4^ events gated for the forward and side light-scatter characteristics of lymphocytes was measured.

The analysis of CD3^+^ memory T cell subsets was performed using Human Naïve/Memory T Cell Panel and anti-CD8^+^ fluorescent labelled antibodies (BD Pharmingen™, San Jose, USA) according to manufacturer’s protocol. Two different cocktails of antibodies (for CD4^+^ and CD8^+^ cells, respectively) containing Alexa Fluor® 647-labelled Mouse Anti-Human CD197 (CCR7), PerCP-Cy™5.5-labelled Mouse Anti-Human CD4, or PE-labelled CD8, and FITC-labelled Mouse Anti-Human CD45RA, were used. For each sample, the fluorescence signal of at least 5x10^4^ ungated events was measured. Quadrants for the dot plots to determine percentages of each CD4^+^ or CD8^+^ memory T cell subsets were derived using appropriate fluorescence-minus-one (FMO) controls.

The measurement of selected cytokines, namely IL-8, IL-1β, IL-6, IL-10, TNF-α, and IL-12p70 concentrations was performed using BD Cytometric Bead Array (CBA) Human Inflammatory Cytokines Kit (BD Biosciences, San Jose, USA) according to manufacturer’s protocol. For each sample, the fluorescence signal of 2100 events gated for capture beads population was measured. Results were calculated using FCAP Array™ Software (ver. 3.0.1; Soft Flow Hungary Ltd., Pecs, Hungary).

### Statistical analysis

Due to low sample size (N < 30), all data are presented as median (interquartile range, IQR), except for age which is presented as median (min-max). Statistical analysis was performed using STATISTICA (data analysis software system), version 12 software (StatSoft, Inc., 2014). Significance of differences observed between analysed time points (pre-exercise *vs.* post-exercise *vs.* recovery) were calculated using Friedman’s analysis of variance for repeated measures followed by *post-hoc* Dunn’s test with Bonferroni correction. Significance of differences in baseline characteristic of the participants and their cardiorespiratory fitness measures between autumn and spring experiments was calculated using Wilcoxon matched pairs test. Each time, P < 0.05 was considered statistically significant.

It must be pointed out that *post-hoc* analysis for Friedman’s ANOVA do not provide exact P values but only allows estimating if any difference is significant on a given significance (α) level.

## Results

A baseline characteristic of the participants is presented in Table 1.

**Table 1:** A baseline characteristic of the participants

**Characteristic**	**Beginning of preparatory phase (autumn competition round) ** **(N = 14)**	**Beginning of preparatory phase (spring competition round) ** **(N = 14)**	**P**
Age (years)	18 (16 - 21)	18 (16 - 21)	NA
Height (cm)	181 (170 - 192)	181 (170 - 192)	NA
Weight (kg)	72.8 (66.3 - 78.1)	69.5 (64.3 - 73.5)	0.300
BMI (kg/m^2^)	21.6 (21.4 - 23.6)	21.5 (20.5 - 23.3)	0.875
BMR (kJ)	8880 (7514 - 8297)	8598 (7870 - 8883)	0.064
FAT (%)	10.0 (7.1 - 10.6)	10.7 (6.5 - 12.3)	0.124
FAT MASS (kg)	6.3 (4.1 - 7.9)	7.6 (4.7 - 9.9)	0.109
FFM (kg)	63.5 (58.6 - 67.1)	65.5 (57.4 - 70.6)	0.594
TBW (kg)	48.5 (43.9 - 49.8)	46.5 (42.9 - 49.2)	0.615
Training experience (years)	10 (9 - 12)	10 (9 - 12)	0.091
Weekly training volume (hours)	12 (10 - 12)	12 (10 - 12)	NA
The table presents median (interquartile range) values (except for the age, where median (min-max) is presented) characterising the participants. BMI - body mass index. BMR - basal metabolic rate. FAT - percentage of fat. FFM - fat free mass. TBW - total body water. NA - not applicable. The difference was tested using the Wilcoxon matched pairs test. P < 0.05 was considered statistically significant.			

No significant differences in cardiorespiratory values were found during the progressive test until exhaustion preformed at the beginning of autumn and spring round, respectively (Table 2). This evidenced that the effort set was comparable in both studied time points.

**Table 2:** Cardiorespiratory fitness measures of participants during the progressive test until exhaustion

**Characteristic**	**Beginning of preparatory phase (autumn competition round) ** **(N = 14)**	**Beginning of preparatory phase (spring competition round) ** **(N = 14)**	**P**
VO_2max_ (mL/kg/min)	60.8 (58.4 - 64.4)	62.2 (60.0 - 64.4)	0.551
HR_max_ (beats/min)	203 (189 - 209)	189 (187 - 203)	0.433
AT (beats/min)	165 (155 - 177)	161 (154 - 174)	0.683
RQ	1.06 (1.06 - 1.09)	1.07 (1.06 - 1.09)	0.382
RC	175 (172 - 192)	172 (172 - 192)	0.660
V_E_ (L/min)	146.7 (122.0 - 156.7)	156.4 (133.6 - 167.7)	0.363
MVV (L/min)	184 (172 - 192)	190 (183 - 200)	0.363
MET (mL/kg/min)	17.3 (16.7 - 18.4)	17.8 (17.1 - 18.7)	0.510
Rf	58.0 (56.1 - 67.5)	58.8 (56.8 - 66.7)	0.975
The table presents median (interquartile range) values. VO_2max_ - maximum oxygen uptake. HR_max _- maximum heart rate. AT - anaerobic threshold. RQ - respiratory quotient (volume ratio of emitted CO_2_ to oxygen uptake). RC - respiratory compensation. V_E_ - minute ventilation. MVV - maximal voluntary ventilation. MET - metabolic equivalent. Rf - respiratory frequency. The difference was tested using the Wilcoxon matched pairs test. P < 0.05 was considered statistically significant.			

Our study showed that after the progressive test (post-exercise) the percentage of total lymphocytes (CD45^+^ cells) in peripheral blood of the soccer players was significantly higher than baseline values, regardless the time of the study (autumn or spring, respectively) (P < 0.05). Even though the CD45^+^ cell percentage was higher after the test (in post-exercise blood sampling time point), the percentages of CD3^+^ cells (T lymphocytes) were lower in autumn experiment. On the other hand, significantly higher CD3^+^ cell percentages were found during recovery time in comparison to post-exercise in both autumn (P < 0.001) and spring (P < 0.001) experiments. Moreover, the CD3^+^ cell percentages found during recovery time in both experiments were significantly higher than the baseline (pre-exercise) values (Table 3). The fluctuations of T cells are in line with Th (CD3^+^CD4^+^) and Tc (CD3^+^CD8^+^) lymphocytes’ distribution. It was found in both experiments (autumn and spring, respectively) that the post-exercise medians of Th cell percentages were significantly lower than baseline values in opposite to recovery time values, when these parameters were higher (in comparison to post-exercise values) (P < 0.05) (Table 3). Significantly lower Tc cell percentages after the progressive test (post-exercise) in comparison to recovery time were observed (*post-hoc* P < 0.01 for autumn and *post-hoc* P < 0.001 for spring experiment, respectively). Our study evidenced that changes in total CD3^+^ cell distribution, especially during the recovery time, are most probably related to Th naïve (CD4^+^CD45RA^+^) cells which are higher in peripheral blood of studied participants regardless of the experiment time (autumn or spring, respectively) (Table 3).

**Table 3:** White blood cell count and lymphocyte subsets investigated in both preparatory phases

	**Beginning of preparatory phase ** **(autumn competition round) ** **(N = 14)**					**Beginning of preparatory phase ** **(spring competition round) ** **(N = 14)**				
	**pre-** **-exercise (1)**	**post-** **-exercise** **(2)**	**recovery** **(3)**	**P**	***Post-hoc* P**	**pre-** **-exercise ** **(1)**	**post-** **-exercise** **(2)**	**recovery** **(3)**	**P**	***Post-hoc *P**
WBC (x 10^9^/L)	6.45 (5.80 - 7.00)	9.05 (7.70 - 11.30)	5.80 (5.10 - 6.10)	< 0.001	(2)/(1)(3)	6.20 (5.30 - 7.00)	8.35 (7.90 - 11.40)	6.55 (6.00 - 7.00)	< 0.001	(2)/(1)(3)
LYM (x 10^9^/L)	1.90 (1.80 - 2.20)	3.35 (2.90 - 4.00)	1.85 (1.70 - 2.10)	< 0.001	(2)/(1)(3)	1.50 (1.40 - 1.80)	2.75 (2.50 - 3.40)	1.90 (1.60 - 2.30)	< 0.001	(2)/(1)(3)
LYM (%)	19.7 (18.4 - 24.5)	25.4 (22.4 - 31.0)	17.5 (16.4 - 18.4)	< 0.001	(2)/(1)(3)	15.7 (13.3 - 19.1)	19.3 (17.1 - 22.3)	21.0 (20.1 - 26.9)	0.003	(1)/(2)(3)
T cells (%)	68.3 (64.4 - 72.7)	58.8 (51.8 - 66.6)	71.8 (66.4 - 74.9)	< 0.001	(1)/(2)/(3)	69.2 (65.2 - 74.0)	62.8 (57.9 - 65.8)	72.0 (67.8 - 79.7)	< 0.001	(3)/(1)(2)
Th cells (%)	54.7 (48.2 - 61.9)	52.5 (43.0 - 58.2)	56.3 (51.6 - 64.4)	< 0.001	(2)/(1)(3)	52.5 (43.4 - 61.6)	45.4 (36.7 - 52.2)	55.4 (46.4 - 62.5)	< 0.001	(2)/(1)(3)
Tc cells (%)	32.7 (28.7 - 41.7)	35.2 (32.3 - 41.4)	32.7 (27.2 - 41.9)	0.010	(2)/(3)	36.2 (34.0 - 41.3)	40.6 (36.6 - 44.3)	35.7 (32.6 - 40.0)	0.001	(2)/(3)
Th naïve cells (%)	40.1 (37.7 - 40.5)	38.1 (33.5 - 40.2)	54.6 (53.5 - 58.5)	< 0.001	(3)/(1)(2)	41.8 (37.3 - 43.3)	39.4 (35.7 - 42.2)	58.0 (52.2 - 63.3)	< 0.001	(3)/(1)(2)
Tc naïve cells (%)	34.2 (32.1 - 38.5)	38.1 (33.9 - 42.5)	39.1 (37. 8 - 42.9)	0.109	-	37.9 (33.3 - 46.6)	38.9 (35.0 - 42.2)	38.4 (32.1 - 41.6)	0.147	-
The table presents median (interquartile range) values. WBC - total white blood cell. LYM - lymphocyte count. The analyses were performed before (pre-exercise) and after the progressive test on mechanical treadmill until exhaustion (5 minutes post-exercise and during recovery time, *cca.* 17 hours after the test). The differences observed between analysed time points (pre-exercise *vs.* post-exercise *vs.* recovery) were assessed using Friedman’s analysis of variance for repeated measures followed by *post-hoc* Dunn’s test with Bonferroni correction. P < 0.05 was considered statistically significant. (2)/(1)(3) - Respective values obtained post-exercise are statistically different compared to pre-exercise and in recovery. (1)/(2)/(3) - Respective values obtained pre-, post-exercise and in recovery are statistically different. (3)/(1)(2) - Respective values obtained in recovery are statistically different compared to pre- and post-exercise. (1)/(2)(3) - Respective values obtained pre-exercise are statistically different compared to post-exercise and in recovery. (2)/(3) - Respective values obtained post-exercise are statistically different compared to recovery.										

No significant differences in Tc central memory (Tc_CM_) and effector memory (Tc_EM_) cells in each time point in both experiments were observed. Significant changes were found in Th_CM_ and Th_EM_ cells. Similarly, in both experiments CD4^+^ effector memory cell percentages were lower during recovery time in comparison to both pre- and post-exercise values. On the other hand, CD4^+^ central memory cell percentage in recovery was lower than baseline values in autumn that is in opposite to preparatory phase to spring competition round when this value was significantly (P < 0.05) higher than baseline one (Table 4).

**Table 4:** T lymphocyte subsets investigated in both preparatory phases

	**Beginning of preparatory phase ** **(autumn competition round) ** **(N = 14)**					**Beginning of preparatory phase ** **(spring competition round) ** **(N = 14)**				
	**pre-** **-exercise (1)**	**post-** **-exercise** **(2)**	**recovery** **(3)**	**P**	***Post-hoc* P**	**pre-** **-exercise ** **(1)**	**post-** **-exercise** **(2)**	**recovery** **(3)**	**P**	***Post-hoc *P**
Tc_CM_ (%)	2.4 (1.6 - 2.7)	2.8 (2.5 - 3.6)	2.8 (2.4 - 3.3)	0.145	-	2.6 (1.3 - 5.6)	9.2 (5.9 - 9.9)	5.4 (2.4 - 9.8)	0.005	(1)/(2)
Tc_EM_ (%)	12.2 (10.6 - 12.7)	10.1 (7.0 - 13.3)	11.7 (10.5 - 14.2)	0.355	-	11.2 (7.9 - 14.5)	10.0 (8.9 - 14.2)	10.5 (9.0 - 13.3)	0.880	-
Th_CM_ (%)	6.2 (4.0 - 11.11)	7.6 (3.1 - 11.8)	3.3 (2.1 - 3.9)	0.019	(2)/(3)	6.9 (4.4 - 8.0)	8.0 (4.9 - 9.8)	9.7 (8.4 - 10.0)	0.046	(1)/(3)
Th_EM_ (%)	12.7 (10.0 - 15.3)	12.7 (10.5 - 15.4)	5.3 (3.3 - 6.7)	< 0.001	(3)/(1)(2)	15.5 (13.8 - 18.7)	15.3 (10.2 - 17.3)	5.0 (3.4 - 5.7)	< 0.001	(3)/(1)(2)
Th17 cells (%)	21.6 (18.5 - 25.8)	20.9 (19.3 - 21.3)	29.2 (26.3 - 32.2)	< 0.001	(3)/(1)(2)	17.4 (15.8 - 19.8)	19.0 (18.1 - 19.6)	27.2 (24.6 - 29.2)	< 0.001	(3)/(1)(2)
Treg (%)	4.0 (3.2 - 5.5)	5.9 (4.2 - 8.3)	11.0 (9.8 - 14.1)	< 0.001	(3)/(1)(2)	4.8 (3.7 - 6.8)	5.6 (4.6 - 6.4)	10.6 (7.5 - 12.7)	0.001	(3)/(1)(2)
Th1 cells (%)	22.7 (21.2 - 26.7)	31.9 (28.7 - 33.7)	34.6 (31.2 - 38.2)	< 0.001	(1)/(2)(3)	15.6 (14.4 - 19.6)	25.6 (21.5 - 29.2)	26.7 (25.2 - 28.1)	< 0.001	(1)/(2)/(3)
Th2 cells (%)	14.9 (10.1 - 18.9)	11.5 (9.9 - 12.9)	12.2 (11.2 - 17.2)	0.257	-	16.4 (13.2 - 18.1)	15.6 (11.8 - 17.3)	16.2 (13.0 - 17.9)	0.832	-
Tc_CM_ - central memory cells. Tc_EM_ - effector memory cells. Th_CM_ - Th central memory cells. Th_EM_ - Th effector memory cells. +). Treg - regulatory T cells. The table presents median (interquartile range) values. The analyses were performed before (pre-exercise) and after the progressive test on mechanical treadmill until exhaustion (5 minutes post-exercise and during recovery time, *cca.* 17 hours after the test). The differences observed between analysed time points (pre-exercise *vs.* post-exercise *vs.* recovery) were assessed using Friedman’s analysis of variance for repeated measures followed by *post-hoc* Dunn’s test with Bonferroni correction. P < 0.05 was considered statistically significant. (3)/(1)(2) - Respective values obtained in recovery are statistically different compared to pre- and post-exercise. (1)/(2)(3) - Respective values obtained pre-exercise are statistically different compared to post-exercise and recovery. (1)/(2)/(3) - Respective values obtained pre-, post-exercise and in recovery are statistically different. (1)/(2) - Respective values obtained pre-exercise are statistically different compared to post-exercise. (2)/(3) - Respective values obtained post-exercise are statistically different compared to recovery. (1)/(3) - Respective values obtained pre-exercise are statistically different compared to recovery.										

Additionally, the differentiation of CD3^+^ cells was analysed in our study. The distribution of four CD3^+^CD4^+^ cells subsets, namely Treg, Th1, Th2 and Th17 was determined. Significantly higher Treg (P < 0.001 and P < 0.01 for autumn and spring, respectively) and Th17 (P < 0.01 and P < 0.01 for autumn and spring, respectively) subsets’ percentages in recovery compared to baseline values were observed in both experiments. Significantly higher Th1 cell subsets were found just after the exercise (P < 0.01) and in recovery (P < 0.001) in both experiments (autumn and spring, respectively), while there were no significant changes in case of Th2 cell subsets’ distribution in peripheral blood of studied participants.

To better understand the mechanism of immune response on exhausting effort, selected cytokine concentrations in plasma were determined (Table 5). Interleukin-8, IL-1β, IL-6, IL-10, TNF-α, and IL-12p70 were determined, yet the IL-1β plasma concentrations were below method’s detection limit in all analysed time points. The studied cytokine profiles seem to be in line with abovementioned changes in analysed T cells’ distribution. At the beginning of the study (that is in the autumn experiment) significant changes in analysed cytokines were observed (Table 5). Repetition of the experiment at the beginning of spring competition round revealed that there were no changes in IL-10 levels (Table 5). It was found that the progressive test until exhaustion in spring caused significantly higher IL-6 (P < 0.05) and IL-8 (P < 0.01) immediately after the test (post-exercise) in comparison to their baseline (pre-exercise) values. Additionally, IL-12p70 concentrations were higher in plasma of the studied soccer players during the recovery time compared to pre- (in autumn experiment) or post-exercise (in both experiments) values. TNF-α in recovery during the autumn experiment and post-exercise during the spring experiment were significantly higher than their baseline values (P < 0.001 and P < 0.05 for recovery sample in autumn and post-exercise sample in spring experiment, respectively). Moreover, significantly higher IL-10 concentrations in recovery time were observed yet only in autumn experiment (P < 0.01 as compared to pre-exercise and P < 0.001 as compared to post-exercise values, respectively) (Table 5). It is worth noting that studied profiles of these cytokines are not completely recurrent in both experiments yet the general pattern of changes has been maintained.

**Table 5:** Plasma cytokine profile investigated in both preparatory phases

	**Beginning of preparatory phase** **(autumn competition round)** **(N = 14)**					**Beginning of preparatory phase ** **(spring competition round) ** **(N = 14)**				
	**pre-** **-exercise (1)**	**post-** **-exercise** **(2)**	**recovery** **(3)**	**P**	***Post-hoc* P**	**pre-** **-exercise ** **(1)**	**post-** **-exercise** **(2)**	**recovery** **(3)**	**P**	***Post-hoc *P**
IL-12p70 (pg/mL)	1.23 (1.02 - 1.77)	1.23 (1.06 - 1.46)	4.61 (3.21 - 5.21)	< 0.001	(3)/(1)(2)	2.23 (1.78 - 2.55)	1.47 (1.11 - 2.44)	2.53 (2.22 - 3.87)	0.002	(2)/(3)
TNF-α (pg/mL)	1.24 (0.90 - 1.65)	1.22 (0.99 - 1.46)	3.92 (2.99 - 4.58)	< 0.001	(3)/(1)(2)	0.92 (0.78 - 1.06)	1.73 (1.62 - 1.87)	1.57 (0.47 - 1.94)	0.017	(1)/(2)
IL-10 (pg/mL)	1.61 (1.51 - 1.98)	1.83 (1.41 - 2.13)	5.54 (4.99 - 6.22)	< 0.001	(3)/(1)(2)	0.97 (0.73 - 1.35)	1.23 (0.79 - 1.25)	1.24 (0.97 - 1.59)	0.135	-
IL-6 (pg/mL)	1.68 (1.30 - 1.79)	1.37 (1.03 - 1.76)	3.01 (2.47 - 4.55)	< 0.001	(3)/(1)(2)	2.01 (1.57 - 2.36)	6.80 (6.45 - 7.49)	12.69 (11.92 - 13.67)	< 0.001	(2)/(1)(3)
IL-8 (pg/mL)	3.62 (3.00 - 4.60)	5.28 (3.76 - 6.38)	3.46 (2.63 - 4.75)	0.001	(2)/(1)(3)	3.85 (2.55 - 5.32)	12.18 (10.66 - 13.16)	13.65 (13.21 - 14.21)	< 0.001	(1)/(2)/(3)
IL-12p70 - Interleukin-12p70. TNF-α - Tumour necrosis factor alpha. IL-10 - Interleukin -10. IL-6 - Interleukin-6. IL-8 - interleukin-8. The table presents median (interquartile range) values. The analyses were performed before (pre-exercise) and after the progressive test on mechanical treadmill until exhaustion (5 minutes post-exercise and during recovery time, *cca.* 17 hours after the test). The differences observed between analysed time points (pre-exercise *vs.* post-exercise *vs.* recovery) were assessed using Friedman’s analysis of variance for repeated measures followed by *post-hoc* Dunn’s test with Bonferroni correction. P < 0.05 was considered statistically significant. (3)/(1)(2) - Respective values obtained in recovery are statistically different compared to pre- and post-exercise. (2)/(1)(3) - Respective values obtained post-exercise are statistically different compared to pre-exercise and in recovery. (1)/(2)/(3) - Respective values obtained pre-, post-exercise and in recovery are statistically different. (1)/(2)/(3) - Respective values obtained pre-, post-exercise and in recovery are statistically different. (1)/(2) - Respective values obtained pre-exercise are statistically different compared to post-exercise. (2)/(3) - Respective values obtained post-exercise are statistically different compared to recovery.										

## Discussion

We have evidenced in our study that maximal effort (100% of VO_2_max) induced increase in total lymphocyte percentage and counts immediately after exercise (Table 3). On the other hand, a significant decrease in T cells was found as a response of athletes’ organisms to exercise in participants’ peripheral blood in autumn experiment. Our results are not in line with other studies where post-effort lymphocytopenia (decrease in blood lymphocyte count below the values found before exercise) occurring usually 0.5 - 1 hour after the exercise was described (*19*-*24*). It may be related with different type of haematological analyses as well as time of blood sampling. It was found in our study that changes in T cells’ distribution are related with post-effort decrease in Th and increase in Tc cell subsets. Additionally, we postulate that general T cells’ percentage was increasing during recovery time in both experiments because the Th cells’ percentage was increased about 5% in comparison to baseline values.

In response to immune system disorders induced by various antigens, including viral or bacterial pathogens, naïve CD4^+^ T cells can differentiate into distinct effector T helper cell lineages capable of differentially regulating the immune response (*5*, *15*). The effector function of newly activated, developed and polarized CD4^+^ T cells depends on the environment where it takes place to match their function to the inflammation agents/pathogens faced (*15*). The observed in our study increase in T cells, including Th ones, in recovery is most likely related with the release of naïve Th cells into peripheral blood. It was not evidenced that maximal effort caused significant changes in peripheral distribution of naïve Tc cells post-exercise as well as in recovery time, respectively in these experiments. The results of our investigations suggested that maximal effort induced changes similar to those induced by well described antigens - viral or bacterial ones. Moreover, we found that the progressive test induced a pro-inflammatory cytokines (IL-12p70 and IL-6) release at the beginning of autumn as well as spring competition round among studied soccer players. These signalling molecules are known as Th1 cell induction promotors (*5*, *15*). These observations are in line with the increase in Th1 cell subset percentages found in both experiments in each blood sampling time point as compared to appropriate pre-exercise value. We also did not found significant changes in Th2 cell subset distribution. These results suggest that the progressive test induces immune response by Th1 signalling pathways and most likely it is not related with the increasing volume of training loads during typical 12-month-long soccer training macrocycle. Similar observations were also found in case of Th17 and Treg cells. The increase in IL-6 plasma concentration is one of the most probable promotor of Th17 differentiation (*11*, *15*).

CD4^+^ regulatory T cells express the transcription factor FoxP3 and are critical in the prevention of excess immunopathology or autoimmunity through multiple mechanisms (*25*). It seems that the increase in CD4^+^ Treg cells observed in our study could be explained by a post-effort inflammation silencing mechanism that might be promoted by Th17 cells. This hypothesis seems to be confirmed by a well-known fact that effector Treg cells appear to be a part of the transcriptional network of effector CD4^+^ T cells in order to match their suppressive function to their present environment (*15*). Knowing that IL-8 and IL-6 are cytokines responsible for haematopoiesis stimulation, the increase in their concentrations found in each time point of both experiments (autumn and spring) may be functional explanation of releasing naïve CD4^+^ T cells into circulation (*26*). Additionally, Treg cells have recently been identified in the muscles where they promote muscle tissue repair through secretion of the growth factor amphiregulin (*25*).

The main limitation of the study is low number of subjects. We have excluded goalkeepers form the experiment because of different training loads. Few participants withdraw their consent to take part in the study or did not take part in the second experiment.

Taking the sleep, diet, infection/illness of the participants into account, the participants are under the continuous care of club dietician and physician being responsible for *e.g.* a proper diet adapted to individual energy requirements, minimum sleep time. Based on that, we assumed that the effect of variables affecting the results would be minimized as much as possible.

We conclude that the progressive test of high intensity (100% VO2max) performed by soccer players caused a significant release of CD4^+^ T naïve cells into circulation. It seems that the movement and a given effort in the progressive test during our study protocol induced an anabolic effect being related not only with cytokine profile but also with CD4^+^ T cells’ differentiation and peripheral distribution. Our findings combined with literature data may suggest that Treg and Th1 cells might be the key important T cell subsets involved in anabolic effect of physical effort.
